# The design of a low-cost 3D printed flow cell for synchrotron computed microtomography

**DOI:** 10.1107/S1600577526000123

**Published:** 2026-02-09

**Authors:** Liam Perera, Peter Garland, Caroline Kirk, Alberto Leonardi, Jason B. Love, Tristan Manchester, Carole A. Morrison, Rebecca Rae, Susanna S. M. Vance, Sharif I. Ahmed

**Affiliations:** a Diamond Light Source Harwell Science and Innovation Campus Didcot OxfordshireOX11 0DE United Kingdom; b School of Chemistry University of Edinburgh Joseph Black Building, Kings Buildings EdinburghEH9 3FJ United Kingdom; University of Malaga, Spain

**Keywords:** synchrotron microtomography, flow cells, 3D printing, diffraction, *in situ*cells, X-ray imaging

## Abstract

This work presents the design and development of a 3D printed flow cell tailored for X-ray computed microtomography of liquid–solid systems. The flow cell is manufactured using stereolithographic printing and utilizes a novel pillarless pull-through geometry. The use of the flow cell developed for K-11 DIAD (Dual Imaging and Diffraction beamline, Diamond Light Source, UK) is demonstrated with the *in situ* flow and selective recovery of an Sn precipitate from solution using an organic ligand. The 3D designs and components are made freely available with this publication.

## Abstract 2

We present and demonstrate the use of a low-cost 3D printed flow cell for the study of liquid–solid systems using synchrotron computed microtomography. The designs are freely available with this publication.

## 1. Introduction

Next-generation light sources are increasing the capacity for high resolution time-resolved X-ray computed microtomography (µCT) and are enabling the study of dynamic processes *in situ*. Sample environments are critical to exploiting this capability. Flow cells are essential tools in synchrotron-based X-ray experiments, facilitating the *in situ* and *operando* study of liquid physio-chemical processes. Flow cells have been used to study a wide range of science areas including, among others, electrochemistry (O’Connor *et al.*, 2022 ▸; Jervis *et al.*, 2016 ▸), hydro­geology (Fusseis *et al.*, 2014 ▸; Thaysen *et al.*, 2025 ▸), low-grade metamorphism and deformation (Freitas *et al.*, 2024 ▸), and nucleation and crystal growth (Yuan *et al.*, 2023 ▸).

Multiple engineering challenges exist in the design of effective flow cells for tomographic imaging (Pak *et al.*, 2023 ▸). These challenges include the need to maximize X-ray transparency of the containment while balancing mechanical integrity and stability, managing beam-induced heating of the liquid and formation of bubbles, and ensuring the system is safe and easy to use. Furthermore, highly customized geometries are required to facilitate integration with beamline-specific equipment such as stages, detectors or complementary optical probes. These challenges result in effective flow cells that are highly specific to the system they are built for, requiring bespoke engineering at the cost of flexibility and price.

Stereolithographic (SLA) 3D printing has emerged as a versatile solution to these challenges. SLA enables rapid prototyping of highly customized monolithic flow cells with complex internal geometries. The additive approach allows faster design iterations and reduces the cost and fabrication time compared with traditional machining. The wide range of available UV-curable resins allows tailoring of material properties to suit the needs of the experiment, and their low-*Z* elemental composition and low density make SLA resins semi-transparent to X-rays. Finally, low-cost bespoke solutions allow for direct integration with beamline-specific sample environments, streamlining the experimental setup and enhancing reproducibility.

In this article, we take advantage of high-resolution SLA 3D printing technology to develop an innovative flow-cell design for µCT applications. We present our design and make it freely available with this publication (details given in the supporting information). The example here was demonstrated on DIAD (Dual Imaging and Diffraction beamline) at Diamond Light Source, UK.

## 2. The DIAD flow cell

**Figure 1:**
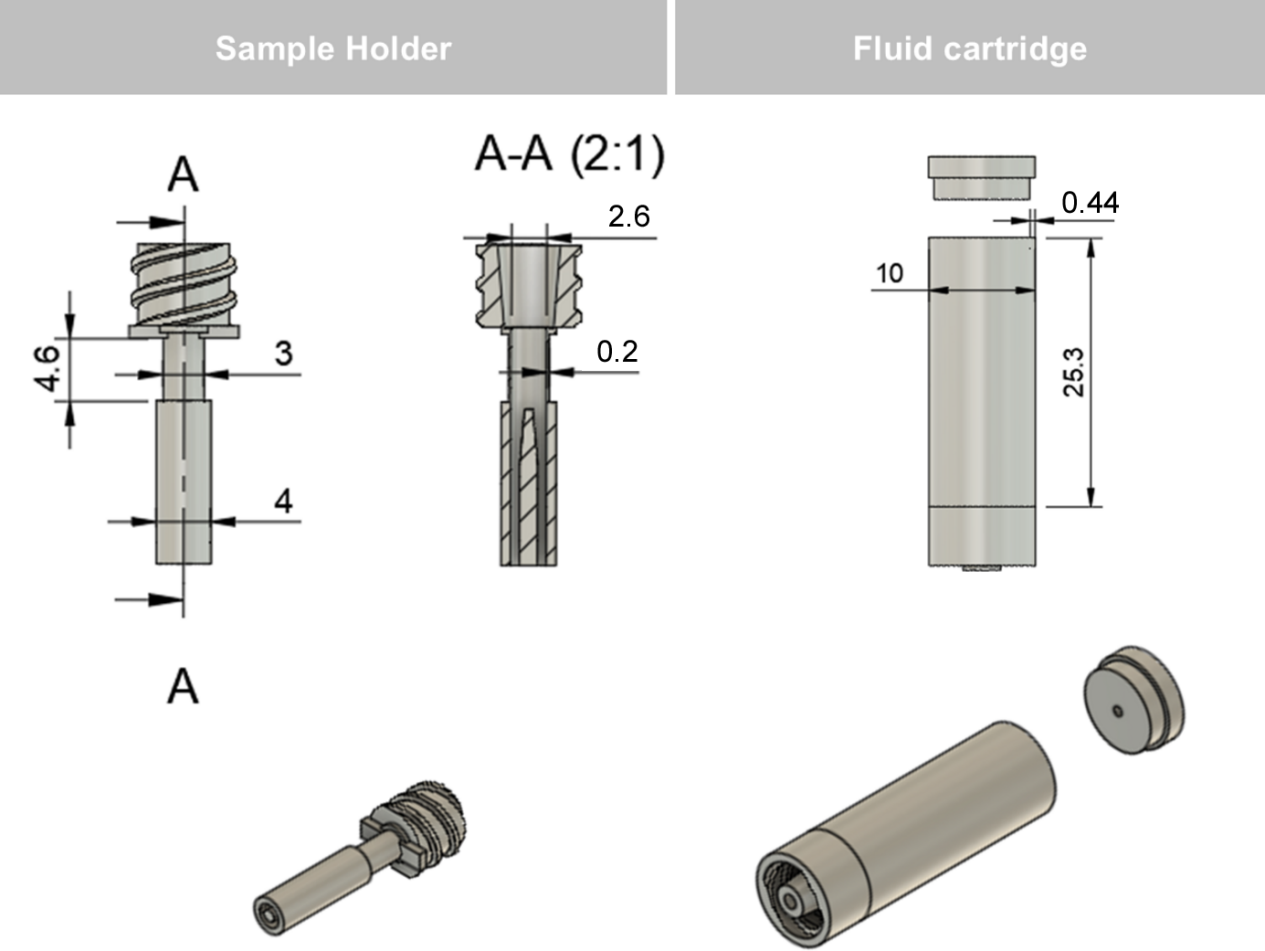
Drawings of (left) the sample holder cell with cut-out and (right) the cartridge with lid, designed and composed in *Fusion360* (Autodesk, 2025 ▸). Components are designed with Luer-lock fittings for leak-tight attachment. Measurements in millimetres.

**Figure 2:**
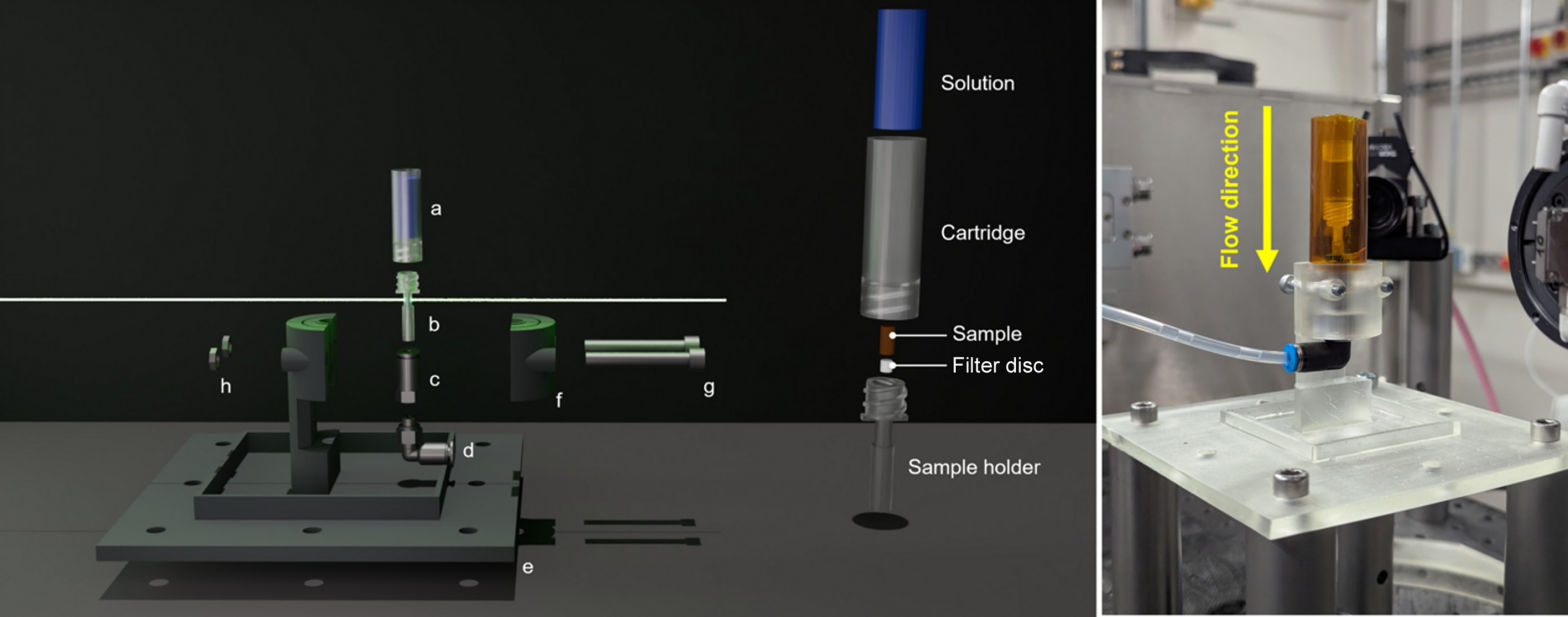
(Left) Exploded rendered view of the flow cell, showing the path of the beam through the sample and components. (*a*) Liquid sample cartridge, (*b*) solid sample holder, (*c*) Festo 4 mm push-fit fitting, (*d*) Festo 4 mm L-fitting rotary union, (*e*) base plate, (*f*) clamp, (*g*) M4 × 35 mm bolt and (*h*) M4 nut. (Centre) Sample and solution loading shown in scaled-up exploded render. (Right) Image of the set up for a flow-cell experiment on DIAD. A Kapton cowl was included as an additional protective measure for any leakage during testing.

The design we present in this paper consists of a 3D SLA-printed disposable sample cell and a reusable cell holder. The component list can be found in the supporting information. The 3D printed sample cell consists of two parts. The first part, the sample holder (Fig. 1 ▸, left), is a hollow tube with a 4 mm base (for push fit) and a female Luer-lock at the top. The inside diameter of the sample holder is 2.6 mm, accommodating approximately 25 mm^3^ of solid sample. The sample cell is optimized for powdered samples which are loaded as a powder into the sample cell on a 2.5 mm filter disc (Sartorius) that is itself supported by a central integrated column that allows lateral flow (Fig. 2 ▸, left). Alternative sample types such as large single grains or monolithic samples could also be used with the current design.

DIAD’s imaging and diffraction field of view (1.4 mm × 1.2 mm) coupled with the energy/flux capability of the beamline constrain the sample diameter (2.6 mm) to ensure good quality imaging and diffraction data. The cell wall is 0.2 mm thick, giving a total cell path length of 0.4 mm through the centre. At 18 keV we calculate the linear attenuation coefficient to be 1.41 cm^−1^, giving an approximate transmission of ∼95% of the incoming beam. This thickness balances the strength of the cell, print quality and consistency, and low X-ray attenuation.

The second component of the cell is a fluid cartridge (Fig. 1 ▸, right) which has a male Luer lock at its base to connect to the sample holder and has capacity for 1.3 ml of fluid for flow-through. The Luer-lock fittings are leak-tight and universal, allowing their use with multiple existing flow fittings. An additional Luer-barb fitting could also be added to the top of the cell to allow for continuous flow, but additional support is required for this geometry.

The cell was designed for a pull-through geometry to ensure that the back pressure could not exceed atmospheric pressure. This was implemented for use with potentially hazardous materials to mitigate the risk of explosive failure from blockages. Flow is initiated using a beamline-integrated syringe pump (Harvard PHD Ultra) that can be operated remotely and can be fully synchronized with scan collection. The fluid flows from the top of the sample to the bottom, where it exits into the outflow tubing (Fig. 2 ▸, right). The proximity of the fluid cartridge allows for instantaneous wetting of the sample once flow is initiated, reducing the need for filling time.

The cell–cartridge combination is attached to the first 4 mm push-fit to M5 female adapter, which itself is coupled to an M5 male to 4 mm push-fit rotary union. The outflow tubing is secured to a guiding post to ensure that the rotary union can move freely. These components are then clamped to a 3D printed base plate with a clamping fixture (Fig. 2 ▸). The use of push-fit fittings allows for easy sample change over, which minimizes the risk of leakage and contamination of surrounding equipment.

The base plate was designed to mount on DIAD’s tomography stage using eight M6 holes pitched 50 mm apart around the edge of the plate. The holder is clamped to the base plate using two 35 mm M4 screws. To accommodate the need for tubing, a rotary union was used downstream of the sample to ensure free rotation of the cell. With such a lightweight sample holder the use of tubing can induce torque and sample movement, both of which act to reduce the quality of tomographic reconstruction. The rotary and holder union allows for approximately 190° of free rotation, providing sufficient freedom for 180° tomography. Full 360° tomography can be achieved with pre-wrapping of the outflow tubing to prevent torque at higher rotation angles. The rotary union also allows the use of more rigid outflow tubing if the experimental conditions require. The 4 mm push-fit represents the smallest diameter available for off-the-shelf rotary unions. Due to the small internal diameter of the sample cell, larger diameter tubing would reduce the flow rate resolution. The 3D printed base plate and clamp have a groove for attaching a Kapton cowl for initial leak containment and an integrated 40 ml drip tray.

Monochromatic energies are required when imaging solid–liquid systems due to beam-induced heating and the formation of bubbles, which can induce motion artefacts in the reconstruction. Monochromatic energy collections are reduced in flux compared with pink-beam collections, meaning the reconstructions are more sensitive to pillars and thicker containment. The cartridge has a fixed volume of solution and allows the cell to be self-supporting without the need for pillars. Pillars or thicker containment are often required to stabilize the sample and support in-flow tubing while the sample is rotating. The consequence is that the signal through the sample is reduced when the pillars rotate into the beam and the contaminated frames reduce the reconstruction quality. Thus, by using a pillarless system we remove the risk of projection contamination and improve the quality of imaging.

The 3D printed components were printed using an SLA printer (Formlabs 3B) with methacrylate clear resin (V4.1). Sample cell components were printed using a layer thickness of 0.05 mm to ensure leak-tight fitting of Luer components. The base required 157 ml of resin, and a batch of 30 cells and cartridges required 47 ml of resin, which amounts to a cost of GBP20 for the total required volume of Formlabs clear resin V4.1. This low cost of printing materials allows for iterative design, with adjustments achievable and testable within the timescale of a user experiment (hours). Mass printing of multiple components allows for the cell to be shipped to the user prior to the experiment, and the option to treat the cell components as disposable allows for their use with potentially hazardous materials. The clear resin allows for remote observation of the cell and flow behaviour during the scan due to its transparency. Additionally, it is stable under extremes of both low and high pH over the timescales of a single experiment (Formlabs, 2024 ▸). Alternative resins could be used to suit the requirements of a different experiment, such as the use of high-temperature resins for flow-through at elevated temperatures.

## 3. Combined synchrotron µCT and XRD

The flow cell was designed for and tested on K11-DIAD at Diamond Light Source, UK (Reinhard *et al.*, 2021 ▸). K11 is a dual-beam instrument with the diffraction and imaging beams configured independently but registered spatially. This allows for image-guided diffraction which is powerful in characterizing heterogenous multiphase systems. The flow cell was commissioned successfully for an *in situ* investigation into base and precious metal recovery from aqueous electronic waste streams. It has been shown previously that the diamide ligand *L* can precipitate metals such as tin as its hexachlorido­metallate complex [(H*L*)_2_SnCl_6_] from HCl solutions (Kinsman *et al.*, 2021 ▸; Vance *et al.*, 2024 ▸). As such, this reaction was probed in the flow cell by combined synchrotron µCT and X-ray diffraction (XRD) on DIAD.

An aqueous acidic solution of Sn (0.01 *M* SnCl_4_ in 6 *M* HCl) was pulled through a powdered sample of a diamide ligand (*L*) over the course of 2 h. The sample was pre-wetted with the metal solution during mounting to improve powder packing and to inhibit the subsequent movement of air bubbles that occurred when the liquid was introduced to the dry sample. Flow was managed with a 5 ml syringe attached to a syringe pump (Harvard PHD Ultra). Scans were taken every 50 µl, with the flow paused during the scan to ensure stability of the sample. The imaging beam was configured to 18 keV with tomograms collected using 1100 projections over 180° with a 0.5 s exposure (9.5 min total). Projections were collected using a PCO edge 5.5 CLHS detector binned four times with a 180 µm GGG:Tb scintillator. The field of view was 1.4 mm × 1.2 mm, giving a final voxel size of 2.16 µm. Binning allowed for shorter scan times, ensuring sufficient time resolution to capture the reaction process. Reconstructions were carried out using filtered back projection with the *ASTRA* toolbox via *Savu* (Wadeson & Basham, 2016 ▸; Vo *et al.*, 2014 ▸; van Aarle *et al.*, 2016 ▸). Diffraction scans were collected using a 25 µm × 25 µm beam at 21 keV. For each flow step, a vertical line scan of ten points was collected and the summed results of ten points per step.

**Figure 3:**
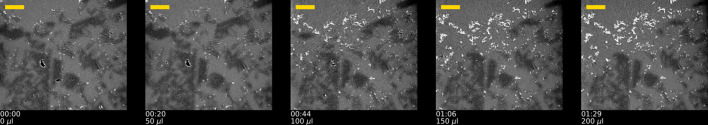
Series showing dissolution precipitation of Sn-containing precipitate during a single flow-through experiment. Sequential *XZ* slices are taken from the centre of the reconstructed volume, with time in minutes and volume in microlitres labelled. Dark features are the precursor ligand and brighter regions represent more-attenuating metal precipitate. The scale bar is 200 µm.

**Figure 4:**
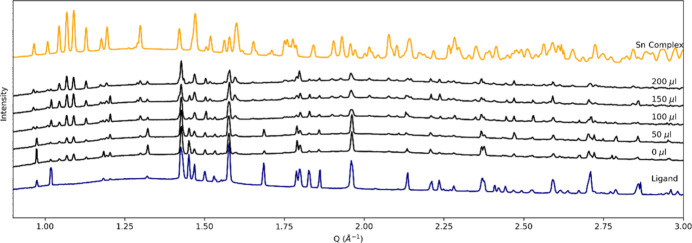
Summed diffraction patterns of (H*L*)_2_[SnCl_6_](H_2_O)_2_ collected at each stage of the flow experiment, demonstrating *in situ* formation of the metal–organic complex.

Successive tomography scans (Fig. 3 ▸) revealed the formation of clusters of a highly absorbing (bright) phase which continued to grow with addition of the metal solution. The combined diffraction (Fig. 4 ▸) identified these clusters as (H*L*)_2_[SnCl_6_](H_2_O)_2_, the formation of which is most obvious in the low-angle peaks (*Q* of 1–1.5 Å^−1^). The artefact-free tomographic reconstruction confirms the mechanical stability of the cell. Furthermore, the clear V4 resin showed good stability over the course of the experiment and exhibited no observable deformation over the course of the 2 h experiment.

## 4. Conclusion

In this work we have presented and demonstrated a low-cost 3D printed flow cell for the study of fluid flow and solid–liquid systems using synchrotron µCT. The simple design of the cell allows for easy modification to suit different sample and experimental conditions and, with the use of SLA printing, it can be printed and rapidly prototyped. The low cost allows cells to be single-use, ideal for use with hazardous samples. We make the design of this cell freely available with this publication (supporting information).

## Supplementary Material

Table S1 and additional 3D printing details. DOI: 10.1107/S1600577526000123/vl5053sup1.pdf

Five stl files for 3D printed flow-cell components. DOI: 10.1107/S1600577526000123/vl5053sup2.zip

## Data Availability

All designs are made freely available with this publication as .stl files.

## References

[bb222] Autodesk (2025). *Fusion.* Autodesk Inc. San Francisco, CA, USA.

[bb1] Formlabs (2024). *Clear Resin V4.1*, https://media.formlabs.com/m/300a539932956deb/original/-ENUS-Clear-Resin-V4-1-TDS.pdf.

[bb2] Freitas, D., Butler, I. B., Elphick, S. C., Gilgannon, J., Rizzo, R. E., Plümper, O., Wheeler, J., Schlepütz, C. M., Marone, F. & Fusseis, F. (2024). *J. Synchrotron Rad.* **31**, 150–161. 10.1107/S1600577523009876 PMC10833432 38117694

[bb3] Fusseis, F., Steeb, H., Xiao, X., Zhu, W., Butler, I. B., Elphick, S. & Mäder, U. (2014). *J. Synchrotron Rad.* **21**, 251–253. 10.1107/S1600577513026969 24365944

[bb4] Jervis, R., Brown, L. D., Neville, T. P., Millichamp, J., Finegan, D. P., Heenan, T. M. M., Brett, D. J. L. & Shearing, P. R. (2016). *J. Phys. D Appl. Phys.* **49**, 434002.

[bb5] Kinsman, L. M. M., Ngwenya, B. T., Morrison, C. A. & Love, J. B. (2021). *Nat. Commun.* **12**, 6258. 10.1038/s41467-021-26563-7 PMC8556376 34716348

[bb6] O’Connor, H., Bailey, J. J., Istrate, O. M., Klusener, P. A. A., Watson, R., Glover, S., Iacoviello, F., Brett, D. J. L., Shearing, P. R. & Nockemann, P. (2022). *Sustain Energy Fuels* **6**, 1529–1540.

[bb7] Pak, T., Archilha, N. L., Berg, S. & Butler, I. B. (2023). *Tomogr. Mater. Struct.* **3**, 100017.

[bb8] Reinhard, C., Drakopoulos, M., Ahmed, S. I., Deyhle, H., James, A., Charlesworth, C. M., Burt, M., Sutter, J., Alexander, S., Garland, P., Yates, T., Marshall, R., Kemp, B., Warrick, E., Pueyos, A., Bradnick, B., Nagni, M., Winter, A. D., Filik, J., Basham, M., Wadeson, N., King, O. N. F., Aslani, N. & Dent, A. J. (2021). *J. Synchrotron Rad.* **28**, 1985–1995. 10.1107/S1600577521009875 PMC8570216 34738954

[bb9] Thaysen, E. M., Butler, I. B., Hassanpouryouzband, A., Spurin, C., Freitas, D., Rizzo, R., Alvarez-Borges, F., Atwood, R. & Edlmann, K. (2025). *J. Colloid Interface Sci.* **694**, 137704. 10.1016/j.jcis.2025.137704 40318288

[bb10] van Aarle, W., Palenstijn, W. J., Cant, J., Janssens, E., Bleichrodt, F., Dabravolski, A., De Beenhouwer, J., Joost Batenburg, K. & Sijbers, J. (2016). *Opt. Express* **24**, 25129–25147. 10.1364/OE.24.025129 27828452

[bb11] Vance, S. S. M., Mojsak, M., Kinsman, L. M. M., Rae, R., Kirk, C., Love, J. B. & Morrison, C. A. (2024). *Inorg. Chem.* **63**, 9332–9345. 10.1021/acs.inorgchem.4c01279 PMC11110006 38722710

[bb12] Vo, N. T., Drakopoulos, M., Atwood, R. C. & Reinhard, C. (2014). *Opt. Express* **22**, 19078. 10.1364/OE.22.019078 25320994

[bb13] Wadeson, N. & Basham, M. (2016). *arXiv*:1610.08015.

[bb14] Yuan, K., Starchenko, V., Rampal, N., Yang, F., Xiao, X. & Stack, A. G. (2023). *J. Synchrotron Rad.* **30**, 634–642. 10.1107/S1600577523002783 PMC10161885 37067259

