# Supplementary material for: The design of a low-cost 3D printed flow cell for synchrotron computed microtomography
Source: J Synchrotron Radiat. 2026 Feb 9;33(Pt 2):511–5. doi: 10.1107/S1600577526000123 (PMC12948017; doi:10.1107/S1600577526000123)
Supplement: Supplementary file 1 [file s-33-00511-sup1.pdf]

JOURNAL OF
SYNCHROTRON
RADIATION

Volume 33 (2026)

Supporting information for article:

The design of a low-cost 3D printed flow cell for synchrotron computed microtomography

Liam Perera, Peter Garland, Caroline Kirk, Alberto Leonardi, Jason B. Love, Tristan Manchester, Carole A. Morrison, Rebecca Rae, Susanna S. M. Vance and Sharif I. Ahmed

Table S1 Component list and comments

Component	Comment
Fluid cartridge*	Print with max 0.05 mm layer thickness
Sample holder*	Print with max 0.05 mm layer thickness
Lid*	Print with max 0.05 mm layer thickness
Base plate*	Recommend 0.1 mm layer thickness
Clamp*	Recommend 0.1 mm layer thickness
Filter cartridge	2.51 mm Safe-Cone filter (Sartorius)
M4 bolt x 35 mm	×2
M4 nut	×2
Pneumatic rotary union M5 - 4mm push fit	×1 (Festo QSRL-1/8-8)
M5 female to push in 4 mm, threaded-to-tube connection style	×1 (Festo QSMF-M5-4)
4 mm tubing	Composition dependent on experimental conditions

* .stl file provided

S2 List of attached .stl files (in ZIP archive)

Base_plate.stl

Fluid_cartridge.stl

Clamp.stl

Lid.stl

Sample_holder.stl
